# Shared Molecular Mechanisms among Alzheimer’s Disease, Neurovascular Unit Dysfunction and Vascular Risk Factors: A Narrative Review

**DOI:** 10.3390/biomedicines10020439

**Published:** 2022-02-14

**Authors:** Lorenzo Falsetti, Giovanna Viticchi, Vincenzo Zaccone, Emanuele Guerrieri, Gianluca Moroncini, Simona Luzzi, Mauro Silvestrini

**Affiliations:** 1 Internal and Subintensive Medicine Department, Azienda Ospedaliero-Universitaria “Ospedali Riuniti” di Ancona, 60100 Ancona, Italy; vincenzo.zaccone@ospedaliriuniti.marche.it; 2 Neurologic Clinic, Marche Polytechnic University, 60126 Ancona, Italy; giovanna.viticchi@ospedaliriuniti.marche.it (G.V.); s.luzzi@univpm.it (S.L.); m.silvestrini@univpm.it (M.S.); 3 Emergency Medicine Residency Program, Università Politecnica delle Marche, 60121 Ancona, Italy; e.guerrieri93@gmail.com; 4 Clinica Medica, Marche Polytechnic University, 60124 Ancona, Italy; g.moroncini@univpm.it

**Keywords:** Alzheimer’s disease, vascular risk factors, hypertension, type 2 diabetes mellitus, dyslipidemia, cigarette smoking

## Abstract

Alzheimer’s disease (AD) is the most common type of dementia, affecting 24 million individuals. Clinical and epidemiological studies have found several links between vascular risk factors (VRF), neurovascular unit dysfunction (NVUd), blood-brain barrier breakdown (BBBb) and AD onset and progression in adulthood, suggesting a pathogenetic continuum between AD and vascular dementia. Shared pathways between AD, VRF, and NVUd/BBB have also been found at the molecular level, underlining the strength of this association. The present paper reviewed the literature describing commonly shared molecular pathways between adult-onset AD, VRF, and NVUd/BBBb. Current evidence suggests that VRF and NVUd/BBBb are involved in AD neurovascular and neurodegenerative pathology and share several molecular pathways. This is strongly supportive of the hypothesis that the presence of VRF can at least facilitate AD onset and progression through several mechanisms, including NVUd/BBBb. Moreover, vascular disease and several comorbidities may have a cumulative effect on VRF and worsen the clinical manifestations of AD. Early detection and correction of VRF and vascular disease by improving NVUd/BBBd could be a potential target to reduce the overall incidence and delay cognitive impairment in AD.

## 1. Introduction

Alzheimer’s disease (AD) is the most common neurodegenerative dementia, affecting two-thirds of individuals with cognitive decline worldwide [1]. Its main pathological features are represented by neuroinflammation, extracellular amyloid-β (Aβ) peptide deposition, intracellular neurofibrillary tangles, tau protein degeneration, and neural loss with progressive deterioration of cognitive function [2,3,4]. Considering a doubling in 20 years, the prevalence of AD will reach 130 million people in 2050, with the greatest increase expected in the poorest countries [5]. Due to its high prevalence, several studies are focusing on reliable serum biomarkers to accurately diagnose AD [3,4].

The neuropathology of AD is characterised by structural and physiological changes that may involve different brain areas. This variability contributes to a certain heterogeneity in the final clinical manifestations in AD patients, each of whom may exhibit a variable association of different neuropsychological deficits. In fact, while the classic cognitive profile of AD is mainly characterised by episodic memory deficits due to the impairment of the temporal lobe, several recent studies have shown that in relation to the different brain areas predominantly involved, different clinical pictures may be present. For example, alterations in the medial prefrontal cortex are associated with impaired retrieval and extinction memories, whereas impairment of emotional and executive processing, similarly to the psychiatric population, reflects a probable impairment of the lateral orbitofrontal cortex or the inferior frontal gyrus [6,7,8]. It is worth underlining that multiple pathways are implicated in the onset and progression of neurodegeneration and specific functions, such as emotional control, are often impaired in dementia. Indeed, impairment of the prefrontal cortex causes in AD patients an impairment in emotion processing that impacts on action and motor control [7].

The AD pathophysiology is considered largely heterogeneous and characterized by both neurodegeneration—characterized by aberrant, misfolded and aggregated Aβ [9] and hyperphosphorylated tau proteins [10]—and vascular disease, with a common involvement of large [11,12,13,14] and small brain vessels. Other neurotoxic elements could also play a relevant role: oxidative stress with reactive oxygen species overproduction, mitochondrial dysfunction or metal accumulation have been extensively studied in the last years [15].

**Figure 1:**
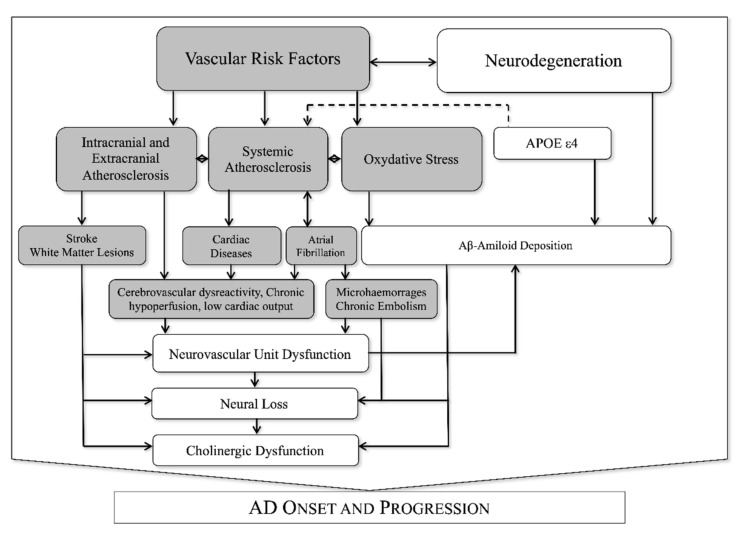
Known interactions between vascular and neurodegenerative factors in AD.

Recently a great attention was put on the interaction between neuronal (neurons and glia) and vascular tissues (endothelial cells, pericytes, and adventitial cells) that are functionally organized in the neurovascular unit (NVU). The NVU is responsible for so-called neurovascular coupling, an organized vascular response to specific neuronal stimuli aimed at modifying regional cerebral blood flow and neuronal metabolic activity [16]. The blood-brain barrier (BBB) is a part of the NVU that controls the transfer of molecules and pathogens to and from brain tissue by adopting a specific transport system in brain endothelial cells [17]. The BBB transports brain metabolic waste products from the brain interstitial fluid to the bloodstream. Thus, BBB represents the most important site of metabolic homeostasis in the central nervous system [17]. Neurovascular coupling is typically deranged in several pathological conditions such as hypertension [18], acute ischemic stroke [19] and AD [20], suggesting a potential role of NVU dysfunction (NVUd) in the progression of cognitive impairment. Of note, neurovascular coupling is strongly influenced by VRF [21,22,23,24,25,26] and atherosclerotic vascular pathology [14,27], as synthesized in Figure 1.

Postmortem studies emphasize an important role of vascular pathology in a large percentage of AD subjects. As first observed in the Nun study [6], the presence of neuropathologic findings of vascular lesions in AD subjects was associated with a history of worse cognitive performances. In addition, other studies have confirmed the presence of atherosclerosis of large and small vessels in AD [12]. These alterations, which could affect NVU activities, may be expressed by pathologic adaptations of cerebrovascular responsiveness to hypercapnia [26,28]. A dysfunction of the BBB has been associated with oxidative stress [29], advanced glycation end products (AGEs) and their receptor (RAGE) [30,31] and increased production of proinflammatory cytokines [32]. A blood-brain barrier breakdown (BBBb) could also contribute to AD onset and progression.

This review focuses on the main and most common vascular risk factors that can be easily detected, monitored, and addressed in common clinical practice, their impact on AD neurovascular and neurodegenerative pathology and the potential links between VRF and AD at a molecular level.

## 2. Research Strategy

The review team identified first the MeSH major terms to explore the association between AD, VRF, NVUd and BBB breakdown and performed the literature search in PubMed/Medline and Web of Science for case reports, reviews, and original research articles from 1 January 1991, to 1 December 2021. We used MeSH major terms and considered: “Alzheimer’s disease” [MeSH], “Adult-onset diabetes mellitus” [MeSH], “Hypertension” [MeSH], “Dyslipidemia” [MeSH], “Cigarette smoking” [MeSH], “Neurovascular coupling” [MeSH], “Blood brain barrier” [MeSH] and “Neurovascular abnormalities” [MeSH] alone or in combination. The review team favored the inclusion of articles from the last 10 years to give up-to-date information, although they did not exclude older highly referenced reports. The reference lists of articles identified by this search strategy were also reviewed, and the working group selected relevant references. We chose to consider this time frame and all types of articles to obtain a comprehensive overview of this topic.

## 3. Discussion of the Results of the Research Strategy

According to the pre-specified research strategy, the review team selected 156 unique papers regarding the clinical, epidemiological and molecular relationships between AD, NVUd, BBBb, type 2 diabetes mellitus (T2DM), hypertension, smoking and dyslipidemia.

### 3.1. Type 2 Diabetes Mellitus

#### 3.1.1. The Clinical and Epidemiological Link between AD and T2DM

Both AD and T2DM prevalence are progressively increasing, especially among elderly patients [1,33]. Among adults, 1 in 11 suffers from diabetes mellitus and 90% of cases are T2DM, which is a chronic, multi-organ disease characterised by a high burden of co-morbidities and a low quality of life [34]. While aging itself is the strongest risk factor for both AD [35] and T2DM [36], emerging epidemiological data suggest that T2DM and other VRFs may contribute to the pathogenesis of AD directly, in association, or as cofactors [37,38,39]. AD and T2DM are epidemiologically associated as AD patients appear more vulnerable to T2DM [40], and individuals with T2DM show an increased risk of dementia, including AD [41].

The Rotterdam Study confirmed that the presence of T2DM increases the risk of AD [42], and that this association is stronger in patients with a long history of T2DM. This epidemiological relationship has been confirmed in other cohorts [43]. However, despite epidemiological studies suggesting T2DM as a potential risk factor for AD, the demonstration of a complete overlap between the two diseases is lacking. Some authors have associated this effect to different insulin resistance in target organs. Cerebral hyperglycemia [44] in the absence of clinically evident T2DM has been positively associated with accelerated cognitive impairment, even adjusting for other risk factors, including age and macrovascular disease. In addition, patients with AD often exhibit both insulin resistance and insulin insufficiency even when not affected by T2DM. These observations have led to the current concept of AD as a special form of T2DM, defined by several authors as “type 3 diabetes mellitus” (T3DM) [45,46,47]. T3DM refers to insulin/insulin-like growth factor (IGF) deficiency and insulin/IGF resistance in brain tissue [46].

#### 3.1.2. The Role of Insulin Signalling

Insulin/IGF and their receptors are widely expressed in the cortex, hippocampus, and hypothalamus of the human brain. Several investigations support the hypothesis that cognitive impairment in AD might be, at least in part, mediated by insulin resistance and deficiency of the insulin/IGF cascade in the brain [48,49,50]. These mechanisms activate multiple intracellular signalling pathways ensuing in intrinsic tyrosine kinases (iTK) activation starting with ligand binding to cell surface receptors, followed by iTK autophosphorylation and activation [51,52]. iTK phosphorylate IRS molecules [53,54,55], which transmit signals downstream by activating the extracellular/mitogen-activated signal-related kinase (ERK/MAPK) and PI3K pathways and inhibit glycogen synthase kinase 3beta (GSK3). PI3K/AKT/mTOR cascade activation leads to synaptic formation, increased neuronal cell survival [56], regional vasodilation, and regulation of cerebrovascular reactivity in the neurovascular unit [57].

Postmortem studies pointed out that, in brain samples from AD patients, insulin and insulin receptor expression were severely impaired and their levels were inversely proportional to the extent of neurodegeneration [45,58] along with impairment of insulin receptor binding capacity and reduced expression of insulin, IGF-1, IGF2 mRNA and their receptors, with a reduction in the cytosolic level of PI3K p85a and p110a subunits [59]. This was consensual with a tau protein reduction, regulated by insulin/IGF-1.

Decreased choline acetyltransferase (ChAT) expression, typically described in AD, is associated with reduced ChAT colocalization with the insulin/IGF-1 receptor, confirming that neuronal expression of tau and ChAT is regulated by insulin/IGF-1 in the human brain [46,47]. Reduced insulin and poor insulin receptor sensitivity contribute to decreased acetylcholine (ACh), further elucidating a possible biochemical link between diabetes and AD [58]. Thus, insulin resistance and deficiency in the brain could explain, at least in part, the alterations observed in AD, such as cytoskeletal collapse, retraction of neurites, synaptic disconnection, loss of neuronal plasticity, and deficiencies in ACh production. Moreover, T2DM is known to be one of the most important factors for accelerated atherosclerosis [60], and these observations suggest that cerebral vessel atherosclerosis could be another potential link between the two diseases, as confirmed by clinical studies [14].

#### 3.1.3. Shared Molecular Mechanisms between AD and T2DM

Primary biological responses to insulin/IGF include increase in cell growth, survival, energy metabolism and cholinergic gene expression, and inhibition of oxidative stress and of apoptosis. These signalling pathways are activated in different cell types and tissues capable of expressing insulin/IGF receptor. Thus they are virtually universal [55,61,62,63]. Several authors enlightened different abnormalities in IRS-1 phosphorylation (IRS-1p) in AD brains [64]. IRS-1p on tyrosine residues is needed for insulin-stimulated responses, whereas IRS-1p on serine residues was associated to an insulin reduced response, which was consistent with insulin resistance [65].

This pathway modulates the expression of Aβ precursor protein (APP), kinesin, Abelson helper integration site-1 (AHI-1), huntingtin-associated protein-1 (HAP-1), and tau, which are all involved in the neuropathology of AD. Furthermore, neuronal and oligodendroglial cell survival and function are fully linked to the integrity of the insulin/IGF-1 pathway [46,47,49]. Impairment of these metabolic pathways leads to deficits in energy metabolism resulting in increased oxidative stress, mitochondrial dysfunction, activation of proinflammatory cytokines and APP expression. Consequently, reduced expression of neuronal and oligodendroglial specific genes and increased expression of astrocytic and microglial inflammatory genes in AD have been attributed to progressive brain insulin/IGF deficiency and resistance.

Microglial and astrocytic APP mRNA levels are increased in the early stages of neurodegeneration in AD [66]. Microglia activation promotes APP gene expression, cleavage and accumulation. Impairment of insulin/IGF signalling leads to oxidative stress and mitochondrial dysfunction that induces APP gene expression and cleavage, thus resulting in neurotoxicity due to APP-A accumulation. Tau gene expression and tau protein phosphorylation are specifically mediated by this signalling cascade [67,68].

#### 3.1.4. The Role of AGE/RAGE System in AD

Glycosylation is a non-reversible and non-enzymatic reaction that occurs between proteins and glucose and eventually leads to the production of AGEs [69], which is especially observed in subjects affected by complicated T2DM [70]. The presence of AGEs marginally affects cell survival, but can significantly alter neuronal metabolism and thus brain function in several neurodegenerative disorders, such as AD [71,72]. In addition, AGEs can directly induce oxidative stress and promote the release of proinflammatory cytokines, thereby worsening cognitive dysfunction in AD [73]. AGEs and RAGEs colocalize with Aβ, senile plaques, and neurofibrillary tangles. Specifically, the interaction between RAGE and Aβ activates neuroinflammatory signalling pathways, causes the release of reactive oxygen species, and ultimately induces neuronal and mitochondrial dysfunction [31].

However, one large study observed a lack of longitudinal association between AGE-RAGE system dysfunction and dementia, suggesting a potential short-term association or reverse causality [74], thus supporting the need for further studies to explore this association.

### 3.2. Hypertension

#### 3.2.1. The Clinical and Epidemiological Link between AD and Hypertension

Aging is an important risk factor for hypertension, representing one of the epidemiological links between AD and VRF. However, the clinical association between hypertension and AD seems weaker than that with T2DM. Some studies have described this potential association [75,76,77] while others failed to demonstrate any link [78,79]. Papers underlining this epidemiological link have longer follow-up times [75,76,77]. The Rotterdam study pointed out that hypertension preceded the onset of AD by nine years [75], while in the Honolulu-Asia Aging Study, a temporal relationship of 20–26 years was observed [77].

On the other hand, some studies have shown that low blood pressure is also associated with incident dementia, and that blood pressure drops in the preclinical stages of AD, during AD, and consistently with advanced cognitive impairment [80]. Recently, a U-shaped relationship between hypertension and cognition has been confirmed, especially among the elderly [81]. Several authors have suggested that this effect might be mediated by neurodegeneration of brain structures involved in the central regulation of blood pressure (hypothalamus, amygdala, insular cortex, medial prefrontal cortex, locus coeruleus, parabrachial nucleus, pons and medulla oblongata). This hypothesis is supported by studies underlining a direct positive relationship between neuron number in pons or medulla and blood pressure in AD [82]. Further, brain atrophy has been correlated with lower blood pressure in elderly patients, regardless of dementia [83]. In addition, lower blood pressure can result in greater neuronal damage by worsening regional cerebral blood flow regulation by generating local hypoperfusion [84].

Thus, middle-aged hypertension acts as a risk factor for AD before its onset, whereas low blood pressure in the elderly should be interpreted as a consequence of neural loss, especially in advanced AD. Hypertension is known to induce cerebral vascular changes and vascular dementia. Animal models have shown that high blood pressure can also lead to AD-like neuropathology [85,86,87], with accumulation and deposition of Aβ.

#### 3.2.2. Shared Molecular Mechanisms between AD and Hypertension

While the “classical” amyloid hypothesis suggested a cytotoxic accumulation of Aβ in the brain tissue of AD patients due to its overproduction [88], more recent evidence has shown that Aβ accumulation might be more related to an altered clearance of this molecule from the BBB due to NVU dysfunction [89].

One of the pathways linking hypertension to AD is RAGE, which modulates Aβ clearance in the BBB. Its expression is critically increased in endothelial cells and at the level of the AD brain neurovascular unit [90]. Furthermore, in experimental models, its expression is upregulated in cerebral vessels of the cortex and hippocampus after exposure to a hypertensive condition [86]. RAGE acts as a scavenger receptor for Aβ. In the BBB it mediates the passage of Aβ from the blood to the brain. It also stimulates Aβ production [91] and induces tau hyperphosphorylation [92] by activating the GSK-3 cascade. The angiotensin-II type 1 receptor suggests another link between AD and hypertension. In hypertensive subjects, activation of this receptor increased RAGE mRNA expression, suggesting a link between activation of the renin-angiotensin axis and AD progression [93]. RAGE also responds to AGEs, which are elevated in AD, especially in patients with T2DM, and this represents another possible link between VRF and AD.

The other molecular mechanism linking AD to hypertension is low-density lipoprotein receptor-related protein-1 (LRP-1). Most cell types in the neurovascular unit express LRP-1, which is able to maintain the BBB integrity and transport Aβ from the brain to the blood vessels, in a direction opposite to that by RAGE. LRP-1 acts primarily by releasing Aβ from the brain, but its soluble form (sLRP-1) can bind Aβ and remove it from the circulation, reducing its bioavailability. Interestingly, the expression of LRP-1 in endothelial cells of the neurovascular unit is reduced with aging and its activity is mediated by ApoE [94]. In murine models of hypertension, RAGE expression is increased, whereas LRP-1 expression is unchanged, suggesting increased Aβ influx that is not adequately counteracted by increased efflux [95]. Furthermore, the presence of oxidative stress, as commonly observed in association with the presence of VRF, decreases sLRP-1 activity and increases serum levels of Aβ, which negatively correlates with cognition [96].

Finally, hypertensive patients often show increased serum levels of several markers of endothelial damage, such as soluble intercellular adhesion molecule-1 (sICAM-1), soluble vascular cell adhesion molecule-1 (sVCAM-1), and endothelin-1 (ET-1), which might be implicated in the dysregulation of cerebrovascular reactivity in AD and other neurodegenerative diseases by promoting vasoconstriction [97,98,99].

### 3.3. Dyslipidaemia

#### 3.3.1. The Clinical and Epidemiological Link between AD and Dyslipidaemia

Dyslipidaemias are a heterogeneous group of diseases defined as disorders of lipid metabolism that lead, alone or in association with other VRFs, to cerebral and systemic atherosclerosis. The current management of dyslipidaemias is closely dependent on the presence and extent of other VRFs, and current guidelines suggest treatment according to the patient’s overall cardiovascular risk, as assessed by formal scores [100]. The systematic assessment and proper management of cardiovascular risk is leading to a progressive reduction in the incidence of atherosclerosis. However, this disease remains one of the leading causes of mortality and morbidity worldwide [100]. The link between dyslipidaemia and AD has been described at several levels. Epidemiological evidence suggests an association between high serum cholesterol levels and AD, with a potential role for lipids in modulating AD expression. Total cholesterol serum levels appear to be independently associated with increased AD prevalence, with a potential modulation of the effect by ApoE genotype [101]. Similar to hypertension, increased serum total cholesterol in middle age also appears to be strongly associated with the risk of AD, with a 3-fold increase in the likelihood of development, independent of ApoE genotype [102]. High levels of LDL cholesterol (LDL-C) correlate with lower global cognition in the absence of clinical dementia [103], and with more rapid cognitive decline in individuals who will develop AD [104]. Some authors underlined a paradoxically protective effect of increased serum cholesterol levels from dementia in late life [105], underlining the detrimental role of dyslipidaemia in younger subjects [106]. In addition, intracranial and extracranial atherosclerosis, one of the major consequences of inadequately treated dyslipidaemia, is significantly associated with the risk of AD onset and progression [14,107].

#### 3.3.2. Shared Molecular Mechanisms between AD and Dyslipidaemia

Increased serum cholesterol levels are presumed to induce neuronal apoptosis, oxidative stress, and tau hyperphosphorylation [108]. Brain lipid composition appears to be directly involved in APP processing and Aβ production: increased endosomal cholesterol levels appear to unbalance APP processing, thereby promoting the amyloid-genic pathway [109,110]. A cholesterol-rich membrane might also alter the activity of membrane secretases, thus inducing Aβ production [111]. Furthermore, dyslipidemia is thought to be associated with BBB disruption, which is commonly observed in AD [112]. Animal models, particularly low-density lipoprotein receptor (LDL-R) knock-out mice, confirm these observations: dyslipidemia increases the severity of cognitive dysfunction, especially learning and memory, and Aβ-associated neurotoxicity [113].

Recent studies have emphasized a genetic overlap between AD, C-reactive protein, and plasma lipids [114]. Genome-wide association studies emphasize a strong association between dyslipidemia and AD in several genes. ApoE genotype has been confirmed central to this interaction [115]. ApoE is the most abundant apolipoprotein in the human brain whose role is to transport lipids and facilitate brain homeostasis by removing debris from the interstitial fluid of the brain by interacting with endothelial cells, the basement membrane and glia [116]. In AD, APOE promotes Aβ clearance. The efficiency of Aβ clearance through the BBB depends on the activity of transport proteins such as APOE and APOJ, and receptors such as LRP-1 and RAGE. In particular, it has been observed that APOE ε2 and APOE ε3 genotypes bind with high affinity with LRP-1, whereas APOE ε4 binds with LDL-R [117]. The lack of interaction between APOE ε4 and LRP-1 has been associated with reduced cyclophilin A (CypA) inhibition leading to a proinflammatory state and BBB breakdown [118]. This effect appears to be mediated in pericytes by an NFB-dependent matrix metalloproteinase 9 (MMP-9), which disrupts endothelial tight junctions [117,119]. In addition, CypA has been associated with systemic atherosclerosis [117].

Other single nucleotide polymorphisms of genes implicated in lipid metabolism, such as CLU and ABCA7, have been associated with AD, underscoring a strong link between lipid homeostasis and cognitive function [120]. Of note, several genes implicated in the modulation of inflammation, such as CR1, HLA-DRB5 and TREM, have also been identified as associated with AD [120].

### 3.4. Cigarette Smoking

#### 3.4.1. The Clinical and Epidemiological Link between AD and Cigarette Smoking

Some cross-sectional studies, supported by the tobacco industry, reported a lower AD prevalence among smokers [121]. However, when analysing incident cases and controlling for tobacco industry affiliation [122], it was observed that smoking consistently increased the risk for AD and cognitive decline [123]. This increased risk was found in both APOE ε4 allele carriers [124] and non-carriers [125]. Particularly, mid-life smoking was associated to an increased AD risk [126]. Smoking habit shows its detrimental effects in cognition at different levels. Compared to non-smokers, middle-aged, active smokers showed poorer neurocognitive performances in executive domains (processing speed, learning and memory). Such cognitive dysfunctions were associated with a reduced volume and thickness in hippocampal, cortical, and subcortical areas, reduced neuronal and BBB integrity and neurobiological alterations like those found in early-stage AD, with a dose-dependent effect. Elderly, active-smoking subjects showed worse executive functions, processing speed, learning and memory, a greater cortical atrophy and lower grey matter density in specific brain areas when compared to non-smokers. Former smokers showed intermediate abnormalities between smokers and non-smokers. Patients with chronic obstructive pulmonary disease (COPD), which is commonly caused by smoking, often show worse cognitive performances [127] that seem to be partially preserved by long-term oxygen therapy [128]. A midlife COPD diagnosis is associated to an increased risk of a later-life cognitive deterioration [129]. However, COPD and lung function impairment seem to affect only marginally incident AD [130].

#### 3.4.2. Shared Molecular Mechanisms between AD and Cigarette Smoking

The only potential neuroprotective effect of smoking on the brain relies on the finding that nicotine showed neuroprotective activity against glutamate toxicity via α4 and α7 subunits, which can inhibit the neuronal apoptosis process similarly to therapeutic acetylcholinesterase inhibitors [131]. Cigarette smoking, however, has been associated to a downregulation of nicotinic acetylcholine receptors (nAChrs) subunit α7 expression on astrocytes [132] with a reduction of the neuroprotection offered. Furthermore, nicotine strongly affects brain endothelial function, since brain endothelium expresses several nicotinic receptor subunits (α3, α5, α7, β2 and β3) [133]. Nicotine increases BBB permeability by reducing tight junctions expression [134]. The detrimental effects of nicotine on tight junctions’ permeability are worsened by oxidative stress and hypoxia. Moreover, nicotine downregulates NOTCH-4 expression in brain endothelial cells: a reduced NOTCH-4 expression is also associated to BBB breakdown [133]. Chronic cigarette smoking has been associated to an increased Aβ deposition and amyloid burden, tau phosphorylation, neuroinflammation with microglial activation, and plaque formation in a dose-dependent manner [135]. On the other side, it has been demonstrated that different central nervous system cells express nicotinic subunits (α3, α4, α5, α6, α7, β2, β4) in the context of nAChrs with a wide variability of expression within different areas of the brain.

Smoking attitude increases oxidative stress by unbalancing the production of reactive oxygen/nitrogen species and their reduction by natural antioxidants [136,137]. Notably, oxidative damage acts on nucleic acids, proteins and lipid membranes of NVU cells [136,137]. Oxidative stress induces cytokine-mediated activation of inflammation in the NVU, thus inducing neuronal cell death and BBBb.

There is a narrow link between neurodegenerative diseases, as AD, and chronic lung pathologies, as COPD [138]. Chronic brain hypoxia, which is commonly observed in advanced COPD and worsened by the occurrence of significant carotid atherosclerosis, seems to worsen cognition by increasing Aβ deposition and tau hyperphosphorylation [139,140]. Moreover, chronic hypoxia acts on VMSCs by downregulating LRP-1 [140], favouring a hypercontractile, non Aβ-clearing phenotype [141]. Moreover, chronic hypoxia has other detrimental effects on cognition: it activates microglia inducing a proinflammatory state that downregulates Aβ receptors and induces a BBB breakdown [140].

### 3.5. Association between VRF and NVU Dysfunction in AD

Neurovascular imbalance is sufficient to initiate neuronal damage and induce accumulation of Aβ. VRF aggregation leads to atherosclerosis, and both factors can induce NVU dysfunction and BBB disruption. These two alterations are associated with increased entry and defective clearance of neurotoxic compounds into brain tissues and reduced energy metabolites and oxygen delivery to activated areas of the brain resulting in neuronal damage. Furthermore, by regulating small vessel blood flow, neurovascular coupling aims to reduce local thrombosis by balancing pro-thrombotic and anti-thrombotic pathways. Its alteration is associated with increased vascular damage. Different combinations of VRF have been associated with cognitive impairment [26,39], especially in the presence of altered cerebrovascular reactivity [142]. Large vessels atherosclerosis is the most prominent effect of a long-term VRF combination, has been associated with an imbalance in cerebrovascular reactivity and more rapid cognitive deterioration [27,143].

At the cellular level, vascular muscle smooth cells (VMSCs) in AD exhibit a “hypercontractile phenotype,” which appears to be critically involved in the dysregulation of local cerebral blood flow by inducing chronic hypoxia and hypoperfusion that facilitates neural loss [141]. In addition, AD-VMSCs exhibit impaired capacity to clear Aβ, facilitating cerebral amyloid angiopathy, which in turn leads to impaired cerebral hemodynamic adaptability [144].

BBB disruption appears to be associated with increased production and reduced clearance of Aβ, which promotes the accumulation of amyloidogenic molecules, typically present in advanced AD. BBB dysfunction is favoured by genetic traits, such as APOE ε4. APOE ε4 carriers show accelerated pericyte degeneration due to activation of the CypA MMP-9 pathway, which is associated with BBB dysfunction with tight junctions and alteration of core proteins [145]. In addition, APOE ε4 carriers often show impaired cerebrovascular reactivity that could affect cerebral perfusion [38,146,147].

## 4. Conclusions

**Figure 2:**
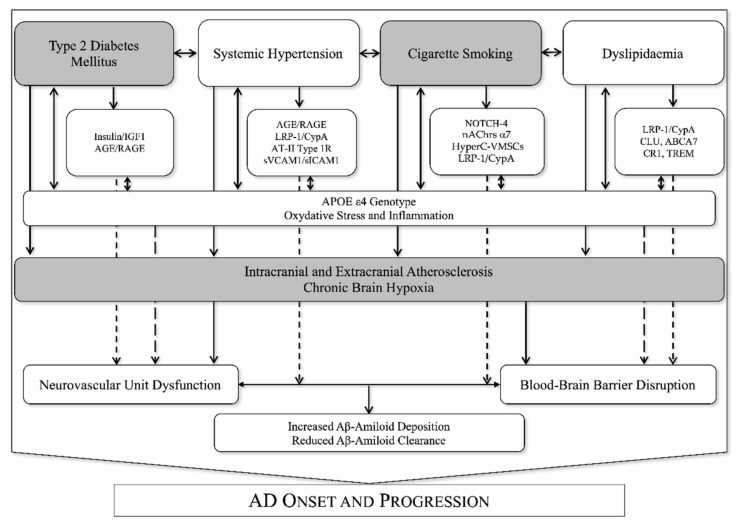
Shared molecular mechanisms linking vascular risk factors, vascular pathology, APOE genotype, neurovascular unit dysfunction, blood-brain barrier dysfunction and Alzheimer’s disease onset and progression. Legend: AGE: advanced glycation end products; AT-II: angiotensin receptor 2; CypA: cyclophilin A; HyperC-VMSCs: hyper-contractile phenotype vascular muscular smoot cells; LRP-1: low-density lipoprotein receptor-related protein-1; nAChrs: nicotinic acetylcholine receptors subunit α7; IGF1: insulin growth factor; RAGE: advanced glycation end products receptor; sICAM1: soluble intercellular adhesion molecule-1; sVCAM1: soluble vascular cell adhesion molecule 1.

This narrative review aimed to focus on the major vascular risk factors that may contribute to AD genesis and progression (as shown in Figure 2). The reviewed literature highlights correlations between VRF, NVU dysfunction, BBB breakdown and AD onset and progression. Older and emerging data suggest data suggest the urgent need for increased attention on VRF detection, monitoring, and correction in all the ageing populations in order to reduce the burden of cognitive deterioration. In addition, these observations suggest—especially in elderly patients—that a global assessment should be carried out, considering ‘classical’ VRF and their aggregation [26]. Moreover, special attention should be paid to various pathological conditions, which are particularly frequent in elderly people such as extracranial and intracranial atherosclerosis [107,148], atrial fibrillation [38,149], chronic lung [129] and kidney [150] disease that could have a detrimental effect on cognition. The strongest link between AD and VRF can be observed in the presence of NVUd and BBB breakdown. In these conditions most of the molecular alterations have been observed. However, although several authors underlined this correlation, less is known on neurovascular unit and blood-brain barrier function after intensive correction of VRF, especially at a molecular level. The current treatment strategy for AD progression has currently focused mainly on correcting neurodegenerative aspects, by also using Aβ-directed monoclonal antibodies [151]. In prospective, especially among elderly, multicomorbid patients with AD, a comprehensive, multi-target approach could be comprehensive not only of an early and intensive VRF correction, but also a of a personalized management of comorbidities in later life to reduce the risk of AD onset and to contain the progression of cognitive impairment also at a vascular level.

## 5. Future Directions

Midlife VRF correction by drugs [152] or physical activity [153,154] has been associated to a reduction of incident dementia, especially AD, and cognitive deterioration in later life. Antihypertensive drugs have already been shown to reduce both the risk and progression of cognitive decline [155]. Oral antidiabetics and insulin seem able to reduce cognitive impairment in AD [156]. Statin use is not associated with an increased risk of cognitive impairment, and some small observational studies seem to associate this treatment with a potentially favourable role in the setting of AD [157]. Long-term oxygen therapy, also, seems to improve cognition hypoxemic patients affected by AD [128]. Recently, 5-phosphodiesterase inhibitors, such as sildenafil, have been shown to improve neurovascular and neurometabolic function in AD [158,159], and are currently under investigation as repurposed drugs for AD treatment by improving NVU function [160]. Analyses of small groups of subjects show that the correction of extracranial carotid stenosis could be associated to an improvement of NVU dysfunction and a reduction of cognitive decline [161]. However, all these observations are largely based on retrospective or non-randomized prospective cohort studies. Larger, robust and long-term trials are required to assess the role of neurometabolic and neurovascular treatment to prevent AD onset and progression. At the present time, in conjunction with the evaluation of the possible benefits of the most modern therapies as Aβ directed treatment or brain stimulation techniques, it could be useful to pay attention to the potential role of carotid surgery or drugs that improve neurovascular and neurometabolic balance [162,163,164].

## Data Availability

Not applicable.

## References

[1] Nichols E., Szoeke C.E.I., Vollset S.E., Abbasi N., Abd-Allah F., Abdela J., Aichour M.T.E., Akinyemi R.O., Alahdab F., Asgedom S.W. (2019). Global, regional, and national burden of Alzheimer’s disease and other dementias, 1990–2016: A systematic analysis for the Global Burden of Disease Study 2016. Lancet Neurol..

[2] Fan L., Mao C., Hu X., Zhang S., Yang Z., Hu Z., Sun H., Fan Y., Dong Y., Yang J. (2020). New Insights Into the Pathogenesis of Alzheimer’s Disease. Front. Neurol..

[3] Török N., Tanaka M., Vécsei L. (2020). Searching for Peripheral Biomarkers in Neurodegenerative Diseases: The Tryptophan-Kynurenine Metabolic Pathway. Int. J. Mol. Sci..

[4] Tanaka M., Toldi J., Vécsei L. (2020). Exploring the Etiological Links behind Neurodegenerative Diseases: Inflammatory Cytokines and Bioactive Kynurenines. Int. J. Mol. Sci..

[5] Alzheimer’s Association (2021). 2021 Alzheimer’s disease facts and figures. Alzheimers Dement..

[6] Battaglia S., Garofalo S., di Pellegrino G. (2018). Context-dependent extinction of threat memories: Influences of healthy aging. Sci. Rep..

[7] Battaglia S., Serio G., Scarpazza C., D’Ausilio A., Borgomaneri S. (2021). Frozen in (e)motion: How reactive motor inhibition is influenced by the emotional content of stimuli in healthy and psychiatric populations. Behav. Res. Ther..

[8] Battaglia S., Harrison B.J., Fullana M.A. (2021). Does the human ventromedial prefrontal cortex support fear learning, fear extinction or both? A commentary on subregional contributions. Mol. Psychiatry.

[9] van der Kant R., Goldstein L.S.B., Ossenkoppele R. (2020). Amyloid-β-independent regulators of tau pathology in Alzheimer disease. Nat. Rev. Neurosci..

[10] Iqbal K., Alonso A.d.C., Chen S., Chohan M.O., El-Akkad E., Gong C.-X., Khatoon S., Li B., Liu F., Rahman A. (2005). Tau pathology in Alzheimer disease and other tauopathies. Biochim. Biophys. Acta Mol. Basis Dis..

[11] Snowdon D.A., Greiner L.H., Mortimer J.A., Riley K.P., Greiner P.A., Markesbery W.R. (1997). Brain infarction and the clinical expression of Alzheimer disease. The Nun Study. JAMA.

[12] Arvanitakis Z., Capuano A.W., Leurgans S.E., Bennett D.A., Schneider J.A. (2016). Relation of cerebral vessel disease to Alzheimer’s disease dementia and cognitive function in elderly people: A cross-sectional study. Lancet Neurol..

[13] Yarchoan M., Xie S.X., Kling M.A., Toledo J.B., Wolk D.A., Lee E.B., Van Deerlin V., Lee V.M.-Y., Trojanowski J.Q., Arnold S.E. (2012). Cerebrovascular atherosclerosis correlates with Alzheimer pathology in neurodegenerative dementias. Brain.

[14] Silvestrini M., Viticchi G., Falsetti L., Balucani C., Vernieri F., Cerqua R., Luzzi S., Bartolini M., Provinciali L. (2011). The role of carotid atherosclerosis in Alzheimer’s disease progression. J. Alzheimers Dis..

[15] Ibrahim M., Gabr M. (2019). Multitarget therapeutic strategies for Alzheimer’s disease. Neural Regen. Res..

[16] Girouard H., Iadecola C. (2006). Neurovascular coupling in the normal brain and in hypertension, stroke, and Alzheimer disease. J. Appl. Physiol..

[17] Zhao Z., Nelson A.R., Betsholtz C., Zlokovic B.V. (2015). Establishment and Dysfunction of the Blood-Brain Barrier. Cell.

[18] Presa J.L., Saravia F., Bagi Z., Filosa J.A. (2020). Vasculo-Neuronal Coupling and Neurovascular Coupling at the Neurovascular Unit: Impact of Hypertension. Front. Physiol..

[19] Silvestrini M., Vernieri F., Pasqualetti P., Matteis M., Passarelli F., Troisi E., Caltagirone C. (2000). Impaired Cerebral Vasoreactivity and Risk of Stroke in Patients With Asymptomatic Carotid Artery Stenosis. JAMA.

[20] Viticchi G., Falsetti L., Vernieri F., Altamura C., Altavilla R., Luzzi S., Bartolini M., Provinciali L., Silvestrini M. (2014). Apolipoprotein E genotype and cerebrovascular alterations can influence conversion to dementia in patients with mild cognitive impairment. J. Alzheimers Dis..

[21] Mogi M., Horiuchi M. (2011). Neurovascular Coupling in Cognitive Impairment Associated With Diabetes Mellitus. Circ. J..

[22] Boms N., Yonai Y., Molnar S., Rosengarten B., Bornstein N.M., Csiba L., Olah L. (2010). Effect of Smoking Cessation on Visually Evoked Cerebral Blood Flow Response in Healthy Volunteers. J. Vasc. Res..

[23] Jennings J.R., Muldoon M.F., Ryan C., Price J.C., Greer P., Sutton-Tyrrell K., van der Veen F.M., Meltzer C.C. (2005). Reduced cerebral blood flow response and compensation among patients with untreated hypertension. Neurology.

[24] Czuba E., Steliga A., Lietzau G., Kowiański P. (2017). Cholesterol as a modifying agent of the neurovascular unit structure and function under physiological and pathological conditions. Metab. Brain Dis..

[25] Hu B., Yan L.-F., Sun Q., Yu Y., Zhang J., Dai Y.-J., Yang Y., Hu Y.-C., Nan H.-Y., Zhang X. (2019). Disturbed neurovascular coupling in type 2 diabetes mellitus patients: Evidence from a comprehensive fMRI analysis. NeuroImage Clin..

[26] Viticchi G., Falsetti L., Buratti L., Luzzi S., Bartolini M., Acciarri M.C., Provinciali L., Silvestrini M. (2015). Metabolic syndrome and cerebrovascular impairment in Alzheimer’s disease. Int. J. Geriatr. Psychiatry.

[27] Buratti L., Balucani C., Viticchi G., Falsetti L., Altamura C., Avitabile E., Provinciali L., Vernieri F., Silvestrini M. (2014). Cognitive deterioration in bilateral asymptomatic severe carotid stenosis. Stroke.

[28] Buratti L., Viticchi G., Falsetti L., Balucani C., Altamura C., Petrelli C., Provinciali L., Vernieri F., Silvestrini M. (2016). Thresholds of impaired cerebral hemodynamics that predict short-term cognitive decline in asymptomatic carotid stenosis. J. Cereb. Blood Flow Metab..

[29] Huang W.-J., Zhang X., Chen W.-W. (2016). Role of oxidative stress in Alzheimer’s disease. Biomed. Rep..

[30] Sasaki N., Fukatsu R., Tsuzuki K., Hayashi Y., Yoshida T., Fujii N., Koike T., Wakayama I., Yanagihara R., Garruto R. (1998). Advanced Glycation End Products in Alzheimer’s Disease and Other Neurodegenerative Diseases. Am. J. Pathol..

[31] Kong Y., Wang F., Wang J., Liu C., Zhou Y., Xu Z., Zhang C., Sun B., Guan Y. (2020). Pathological Mechanisms Linking Diabetes Mellitus and Alzheimer’s Disease: The Receptor for Advanced Glycation End Products (RAGE). Front. Aging Neurosci..

[32] Swardfager W., Lanctôt K., Rothenburg L., Wong A., Cappell J., Herrmann N. (2010). A Meta-Analysis of Cytokines in Alzheimer’s Disease. Biol. Psychiatry.

[33] Khan M.A.B., Hashim M.J., King J.K., Govender R.D., Mustafa H., Al Kaabi J. (2020). Epidemiology of Type 2 Diabetes—Global Burden of Disease and Forecasted Trends. J. Epidemiol. Glob. Health.

[34] Zheng Y., Ley S.H., Hu F.B. (2018). Global aetiology and epidemiology of type 2 diabetes mellitus and its complications. Nat. Rev. Endocrinol..

[35] Guerreiro R., Bras J. (2015). The age factor in Alzheimer’s disease. Genome Med..

[36] Fazeli P.K., Lee H., Steinhauser M.L. (2020). Aging Is a Powerful Risk Factor for Type 2 Diabetes Mellitus Independent of Body Mass Index. Gerontology.

[37] Qiu C., De Ronchi D., Fratiglioni L. (2007). The epidemiology of the dementias: An update. Curr. Opin. Psychiatry.

[38] Falsetti L., Viticchi G., Buratti L., Grigioni F., Capucci A., Silvestrini M. (2018). Interactions between Atrial Fibrillation, Cardiovascular Risk Factors, and ApoE Genotype in Promoting Cognitive Decline in Patients with Alzheimer’s Disease: A Prospective Cohort Study. J. Alzheimers Dis..

[39] Viticchi G., Falsetti L., Buratti L., Boria C., Luzzi S., Bartolini M., Provinciali L., Silvestrini M. (2015). Framingham risk score can predict cognitive decline progression in Alzheimer’s disease. Neurobiol. Aging.

[40] Janson J., Laedtke T., Parisi J.E., O’Brien P., Petersen R.C., Butler P.C. (2004). Increased Risk of Type 2 Diabetes in Alzheimer Disease. Diabetes.

[41] Biessels G.J., Staekenborg S., Brunner E., Brayne C., Scheltens P. (2006). Risk of dementia in diabetes mellitus: A systematic review. Lancet Neurol..

[42] Hofman A., Ott A., Breteler M.M., Bots M.L., Slooter A.J., van Harskamp F., van Duijn C.N., Van Broeckhoven C., Grobbee D.E. (1997). Atherosclerosis, apolipoprotein E, and prevalence of dementia and Alzheimer’s disease in the Rotterdam Study. Lancet.

[43] Kloppenborg R.P., van den Berg E., Kappelle L.J., Biessels G.J. (2008). Diabetes and other vascular risk factors for dementia: Which factor matters most? A systematic review. Eur. J. Pharmacol..

[44] Crane P.K., Walker R., Hubbard R.A., Li G., Nathan D.M., Zheng H., Haneuse S., Craft S., Montine T.J., Kahn S.E. (2013). Glucose Levels and Risk of Dementia. N. Engl. J. Med..

[45] Steen E., Terry B.M., Rivera E.J., Cannon J.L., Neely T.R., Tavares R., Xu X.J., Wands J.R., de la Monte S.M. (2005). Impaired insulin and insulin-like growth factor expression and signaling mechanisms in Alzheimer’s disease—Is this type 3 diabetes?. J. Alzheimers Dis..

[46] de la Monte S.M., Wands J.R. (2008). Alzheimer’s Disease is Type 3 Diabetes—Evidence Reviewed. J. Diabetes Sci. Technol..

[47] de la Monte S.M., Tong M., Lester-Coll N., Plater M., Wands J.R. (2006). Therapeutic rescue of neurodegeneration in experimental type 3 diabetes: Relevance to Alzheimer’s disease. J. Alzheimers Dis..

[48] de la Monte S.M. (2012). Brain Insulin Resistance and Deficiency as Therapeutic Targets in Alzheimers Disease. Curr. Alzheimer Res..

[49] Lester-Coll N., Rivera E.J., Soscia S.J., Doiron K., Wands J.R., de la Monte S.M. (2006). Intracerebral streptozotocin model of type 3 diabetes: Relevance to sporadic Alzheimer’s disease. J. Alzheimers Dis..

[50] de la Monte S.M., Ganju N., Banerjee K., Brown N.V., Luong T., Wands J.R. (2000). Partial rescue of ethanol-induced neuronal apoptosis by growth factor activation of phosphoinositol-3-kinase. Alcohol. Clin. Exp. Res..

[51] Myers M.G., Sun X.J., White M.F. (1994). The IRS-1 signaling system. Trends Biochem. Sci..

[52] Ullrich A., Bell J.R., Chen E.Y., Herrera R., Petruzzelli L.M., Dull T.J., Gray A., Coussens L., Liao Y.-C., Tsubokawa M. (1985). Human insulin receptor and its relationship to the tyrosine kinase family of oncogenes. Nature.

[53] Sun X.J., Rothenberg P., Kahn C.R., Backer J.M., Araki E., Wilden P.A., Cahill D.A., Goldstein B.J., White M.F. (1991). Structure of the insulin receptor substrate IRS-1 defines a unique signal transduction protein. Nature.

[54] White M.F., Maron R., Kahn C.R. (1985). Insulin rapidly stimulates tyrosine phosphorylation of a Mr-185,000 protein in intact cells. Nature.

[55] Sun X.J., Crimmins D.L., Myers M.G., Miralpeix M., White M.F. (1993). Pleiotropic insulin signals are engaged by multisite phosphorylation of IRS-1. Mol. Cell. Biol..

[56] Matsuda S., Nakagawa Y., Tsuji A., Kitagishi Y., Nakanishi A., Murai T. (2018). Implications of PI3K/AKT/PTEN Signaling on Superoxide Dismutases Expression and in the Pathogenesis of Alzheimer’s Disease. Diseases.

[57] Chang C.-Z., Wu S.-C., Chang C.-M., Lin C.-L., Kwan A.-L. (2015). Arctigenin, a Potent Ingredient of *Arctium lappa* L., Induces Endothelial Nitric Oxide Synthase and Attenuates Subarachnoid Hemorrhage-Induced Vasospasm through PI3K/Akt Pathway in a Rat Model. BioMed Res. Int..

[58] Rivera E.J., Goldin A., Fulmer N., Tavares R., Wands J.R., de la Monte S.M. (2005). Insulin and insulin-like growth factor expression and function deteriorate with progression of Alzheimer’s disease: Link to brain reductions in acetylcholine. J. Alzheimers Dis..

[59] Moloney A.M., Griffin R.J., Timmons S., O’Connor R., Ravid R., O’Neill C. (2010). Defects in IGF-1 receptor, insulin receptor and IRS-1/2 in Alzheimer’s disease indicate possible resistance to IGF-1 and insulin signalling. Neurobiol. Aging.

[60] Basta G., Schmidt A.M., De Caterina R. (2004). Advanced glycation end products and vascular inflammation: Implications for accelerated atherosclerosis in diabetes. Cardiovasc. Res..

[61] Burgering B.M.T., Coffer P.J. (1995). Protein kinase B (c-Akt) in phosphatidylinositol-3-OH kinase signal transduction. Nature.

[62] Delcommenne M., Tan C., Gray V., Rue L., Woodgett J., Dedhar S. (1998). Phosphoinositide-3-OH kinase-dependent regulation of glycogen synthase kinase 3 and protein kinase B/AKT by the integrin-linked kinase. Proc. Natl. Acad. Sci. USA.

[63] Kulik G., Klippel A., Weber M.J. (1997). Antiapoptotic signalling by the insulin-like growth factor I receptor, phosphatidylinositol 3-kinase, and Akt. Mol. Cell. Biol..

[64] Talbot K., Wang H.-Y., Kazi H., Han L.-Y., Bakshi K.P., Stucky A., Fuino R.L., Kawaguchi K.R., Samoyedny A.J., Wilson R.S. (2012). Demonstrated brain insulin resistance in Alzheimer’s disease patients is associated with IGF-1 resistance, IRS-1 dysregulation, and cognitive decline. J. Clin. Investig..

[65] Hirosumi J., Tuncman G., Chang L., Görgün C.Z., Uysal K.T., Maeda K., Karin M., Hotamisligil G.S. (2002). A central role for JNK in obesity and insulin resistance. Nature.

[66] de la Monte S.M., Wands J.R. (2006). Molecular indices of oxidative stress and mitochondrial dysfunction occur early and often progress with severity of Alzheimer’s disease. J. Alzheimers Dis..

[67] Schubert M., Brazil D.P., Burks D.J., Kushner J.A., Ye J., Flint C.L., Farhang-Fallah J., Dikkes P., Warot X.M., Rio C. (2003). Insulin Receptor Substrate-2 Deficiency Impairs Brain Growth and Promotes Tau Phosphorylation. J. Neurosci..

[68] Schubert M., Gautam D., Surjo D., Ueki K., Baudler S., Schubert D., Kondo T., Alber J., Galldiks N., Küstermann E. (2004). Role for neuronal insulin resistance in neurodegenerative diseases. Proc. Natl. Acad. Sci. USA.

[69] Bunn H., Higgins P. (1981). Reaction of monosaccharides with proteins: Possible evolutionary significance. Science.

[70] Simó R., Ciudin A., Simó-Servat O., Hernández C. (2017). Cognitive impairment and dementia: A new emerging complication of type 2 diabetes—The diabetologist’s perspective. Acta Diabetol..

[71] Miranda H.V., Outeiro T.F. (2010). The sour side of neurodegenerative disorders: The effects of protein glycation. J. Pathol..

[72] Salahuddin P., Rabbani G., Khan R. (2014). The role of advanced glycation end products in various types of neurodegenerative disease: A therapeutic approach. Cell. Mol. Biol. Lett..

[73] Yan S.D., Chen X., Fu J., Chen M., Zhu H., Roher A., Slattery T., Zhao L., Nagashima M., Morser J. (1996). RAGE and amyloid-β peptide neurotoxicity in Alzheimer’s disease. Nature.

[74] Chen J., Mooldijk S.S., Licher S., Waqas K., Ikram M.K., Uitterlinden A.G., Zillikens M.C., Ikram M.A. (2021). Assessment of Advanced Glycation End Products and Receptors and the Risk of Dementia. JAMA Netw. Open.

[75] Portegies M.L.P., Mirza S.S., Verlinden V.J.A., Hofman A., Koudstaal P.J., Swanson S.A., Ikram M.A. (2016). Mid- to Late-Life Trajectories of Blood Pressure and the Risk of Stroke. Hypertension.

[76] Kivipelto M., Helkala E.-L., Laakso M., Hänninen T., Hallikainen M., Alhainen K., Soininen H., Tuomilehto J., Nissinen A. (2001). Midlife vascular risk factors and Alzheimer’s disease in later life: Longitudinal, population based study. BMJ.

[77] Launer L.J., Ross G.W., Petrovitch H., Masaki K., Foley D., White L.R., Havlik R.J. (2000). Midlife blood pressure and dementia: The Honolulu–Asia aging study☆. Neurobiol. Aging.

[78] Posner H.B., Tang M.-X., Luchsinger J., Lantigua R., Stern Y., Mayeux R. (2002). The relationship of hypertension in the elderly to AD, vascular dementia, and cognitive function. Neurology.

[79] Yoshitake T., Kiyohara Y., Kato I., Ohmura T., Iwamoto H., Nakayama K., Ohmori S., Nomiyama K., Kawano H., Ueda K. (1995). Incidence and risk factors of vascular dementia and Alzheimer’s disease in a defined elderly Japanese population: The Hisayama Study. Neurology.

[80] Skoog I., Nilsson L., Persson G., Lernfelt B., Landahl S., Palmertz B., Andreasson L.-A., Odén A., Svanborg A. (1996). 15-year longitudinal study of blood pressure and dementia. Lancet.

[81] van Dalen J.W., Brayne C., Crane P.K., Fratiglioni L., Larson E.B., Lobo A., Lobo E., Marcum Z.A., van Charante E.P.M., Qiu C. (2021). Association of Systolic Blood Pressure With Dementia Risk and the Role of Age, U-Shaped Associations, and Mortality. JAMA Intern. Med..

[82] Burke W.J., Coronado P.G., Schmitt C.A., Gillespie K.M., Chung H.D. (1994). Blood pressure regulation in alzheimer’s disease. J. Auton. Nerv. Syst..

[83] Skoog I., Andreasson L.-A., Landahl S., Lernfelt B. (1998). A Population-Based Study on Blood Pressure and Brain Atrophy in 85-Year-Olds. Hypertension.

[84] de la Torre J.C. (2000). Cerebral Hypoperfusion, Capillary Degeneration, and Development of Alzheimer Disease. Alzheimer Dis. Assoc. Disord..

[85] Carnevale D., Lembo G. (2011). ‘Alzheimer-like’ pathology in a murine model of arterial hypertension. Biochem. Soc. Trans..

[86] Carnevale D., Mascio G., D’Andrea I., Fardella V., Bell R.D., Branchi I., Pallante F., Zlokovic B., Yan S.S., Lembo G. (2012). Hypertension Induces Brain β-Amyloid Accumulation, Cognitive Impairment, and Memory Deterioration Through Activation of Receptor for Advanced Glycation End Products in Brain Vasculature. Hypertension.

[87] Carnevale D., Mascio G., Ajmone-Cat M.A., D’Andrea I., Cifelli G., Madonna M., Cocozza G., Frati A., Carullo P., Carnevale L. (2012). Role of neuroinflammation in hypertension-induced brain amyloid pathology. Neurobiol. Aging.

[88] Hardy J., Higgins G. (1992). Alzheimer’s disease: The amyloid cascade hypothesis. Science.

[89] Zlokovic B.V. (1996). Cerebrovascular transport of Alzheimer’s amyloidβ and apolipoproteins J and E: Possible anti-amyloidogenic role of the blood-brain barrier. Life Sci..

[90] Deane R., Du Yan S., Submamaryan R.K., LaRue B., Jovanovic S., Hogg E., Welch D., Manness L., Lin C., Yu J. (2003). RAGE mediates amyloid-β peptide transport across the blood-brain barrier and accumulation in brain. Nat. Med..

[91] Chen C., Li X.-H., Tu Y., Sun H.-T., Liang H.-Q., Cheng S.-X., Zhang S. (2014). Aβ-AGE aggravates cognitive deficit in rats via RAGE pathway. Neuroscience.

[92] Li X.-H., Lv B.-L., Xie J.-Z., Liu J., Zhou X.-W., Wang J.-Z. (2012). AGEs induce Alzheimer-like tau pathology and memory deficit via RAGE-mediated GSK-3 activation. Neurobiol. Aging.

[93] Nakamura K., Yamagishi S., Nakamura Y., Takenaka K., Matsui T., Jinnouchi Y., Imaizumi T. (2005). Telmisartan inhibits expression of a receptor for advanced glycation end products (RAGE) in angiotensin-II-exposed endothelial cells and decreases serum levels of soluble RAGE in patients with essential hypertension. Microvasc. Res..

[94] Shibata M., Yamada S., Kumar S.R., Calero M., Bading J., Frangione B., Holtzman D.M., Miller C.A., Strickland D.K., Ghiso J. (2000). Clearance of Alzheimer’s amyloid-β1-40 peptide from brain by LDL receptor–related protein-1 at the blood-brain barrier. J. Clin. Investig..

[95] Shih Y.-H., Wu S.-Y., Yu M., Huang S.-H., Lee C.-W., Jiang M.-J., Lin P.-Y., Yang T.-T., Kuo Y.-M. (2018). Hypertension Accelerates Alzheimer’s Disease-Related Pathologies in Pigs and 3xTg Mice. Front. Aging Neurosci..

[96] Sagare A.P., Deane R., Zetterberg H., Wallin A., Blennow K., Zlokovic B.V. (2011). Impaired Lipoprotein Receptor-Mediated Peripheral Binding of Plasma Amyloid-β is an Early Biomarker for Mild Cognitive Impairment Preceding Alzheimer’s Disease. J. Alzheimers Dis..

[97] Uiterwijk R., Huijts M., Staals J., Rouhl R.P.W., De Leeuw P.W., Kroon A.A., Van Oostenbrugge R.J. (2016). Endothelial Activation Is Associated With Cognitive Performance in Patients With Hypertension. Am. J. Hypertens..

[98] Rosei E.A., Rizzoni D., Muiesan M.L., Sleiman I., Salvetti M., Monteduro C., Porteri E. (2005). Effects of candesartan cilexetil and enalapril on inflammatory markers of atherosclerosis in hypertensive patients with non-insulin-dependent diabetes mellitus. J. Hypertens..

[99] Akter S., Jesmin S., Iwashima Y., Hideaki S., Rahman M.A., Islam M.M., Moroi M., Shimojo N., Yamaguchi N., Miyauchi T. (2015). Higher circulatory level of endothelin-1 in hypertensive subjects screened through a cross-sectional study of rural Bangladeshi women. Hypertens. Res..

[100] Visseren F.L.J., Mach F., Smulders Y.M., Carballo D., Koskinas K.C., Bäck M., Benetos A., Biffi A., Boavida J.-M., Capodanno D. (2021). 2021 ESC Guidelines on cardiovascular disease prevention in clinical practice. Eur. Heart J..

[101] Notkola I.-L., Sulkava R., Pekkanen J., Erkinjuntti T., Ehnholm C., Kivinen P., Tuomilehto J., Nissinen A. (1998). Serum Total Cholesterol, Apolipoprotein E {FC12}e4 Allele, and Alzheimer’s Disease. Neuroepidemiology.

[102] Kivipelto M., Helkala E.-L., Laakso M.P., Hänninen T., Hallikainen M., Alhainen K., Iivonen S., Mannermaa A., Tuomilehto J., Nissinen A. (2002). Apolipoprotein E ϵ4 Allele, Elevated Midlife Total Cholesterol Level, and High Midlife Systolic Blood Pressure Are Independent Risk Factors for Late-Life Alzheimer Disease. Ann. Intern. Med..

[103] Yaffe K., Barrett-Connor E., Lin F., Grady D. (2002). Serum Lipoprotein Levels, Statin Use, and Cognitive Function in Older Women. Arch. Neurol..

[104] Helzner E.P., Luchsinger J.A., Scarmeas N., Cosentino S., Brickman A.M., Glymour M.M., Stern Y. (2009). Contribution of Vascular Risk Factors to the Progression in Alzheimer Disease. Arch. Neurol..

[105] Mielke M.M., Zandi P.P., Sjogren M., Gustafson D., Ostling S., Steen B., Skoog I. (2005). High total cholesterol levels in late life associated with a reduced risk of dementia. Neurology.

[106] Whitmer R.A., Sidney S., Selby J., Johnston S.C., Yaffe K. (2005). Midlife cardiovascular risk factors and risk of dementia in late life. Neurology.

[107] Roher A.E., Esh C., Kokjohn T.A., Kalback W., Luehrs D.C., Seward J.D., Sue L.I., Beach T.G. (2003). Circle of Willis Atherosclerosis Is a Risk Factor for Sporadic Alzheimer’s Disease. Arterioscler. Thromb. Vasc. Biol..

[108] McLaurin J., Darabie A.A., Morrison M.R. (2002). Cholesterol, a Modulator of Membrane-Associated Aβ-Fibrillogenesis. Ann. N. Y. Acad. Sci..

[109] Sun F., Chen L., Wei P., Chai M., Ding X., Xu L., Luo S.-Z. (2017). Dimerization and Structural Stability of Amyloid Precursor Proteins Affected by the Membrane Microenvironments. J. Chem. Inf. Model..

[110] Brown A.M., Bevan D.R. (2017). Influence of sequence and lipid type on membrane perturbation by human and rat amyloid β-peptide (1–42). Arch. Biochem. Biophys..

[111] Abad-Rodriguez J., Ledesma M.D., Craessaerts K., Perga S., Medina M., Delacourte A., Dingwall C., De Strooper B., Dotti C.G. (2004). Neuronal membrane cholesterol loss enhances amyloid peptide generation. J. Cell Biol..

[112] Bowman G.L., Kaye J.A., Quinn J.F. (2012). Dyslipidemia and Blood-Brain Barrier Integrity in Alzheimer’s Disease. Curr. Gerontol. Geriatr. Res..

[113] de Oliveira J., Moreira E.L.G., dos Santos D.B., Piermartiri T.C., Dutra R.C., Pinton S., Tasca C.I., Farina M., Prediger R.D.S., de Bem A.F. (2014). Increased Susceptibility to Amyloid-β-Induced Neurotoxicity in Mice Lacking the Low-Density Lipoprotein Receptor. J. Alzheimers Dis..

[114] Desikan R.S., Schork A.J., Wang Y., Thompson W.K., Dehghan A., Ridker P.M., Chasman D.I., McEvoy L.K., Holland D., Chen C.-H. (2015). Polygenic Overlap Between C-Reactive Protein, Plasma Lipids, and Alzheimer Disease. Circulation.

[115] Coon K.D., Myers A.J., Craig D.W., Webster J.A., Pearson J.V., Lince D.H., Zismann V.L., Beach T.G., Leung D., Bryden L. (2007). A High-Density Whole-Genome Association Study Reveals That APOE Is the Major Susceptibility Gene for Sporadic Late-Onset Alzheimer’s Disease. J. Clin. Psychiatry.

[116] Flowers S.A., Rebeck G.W. (2020). APOE in the normal brain. Neurobiol. Dis..

[117] Bell R.D. (2012). The Imbalance of Vascular Molecules in Alzheimer’s Disease. J. Alzheimers Dis..

[118] Nikolakopoulou A.M., Wang Y., Ma Q., Sagare A.P., Montagne A., Huuskonen M.T., Rege S.V., Kisler K., Dai Z., Körbelin J. (2021). Endothelial LRP1 protects against neurodegeneration by blocking cyclophilin A. J. Exp. Med..

[119] Nigro P., Satoh K., O’Dell M.R., Soe N.N., Cui Z., Mohan A., Abe J., Alexis J.D., Sparks J.D., Berk B.C. (2011). Cyclophilin A is an inflammatory mediator that promotes atherosclerosis in apolipoprotein E-deficient mice. J. Exp. Med..

[120] Karch C.M., Cruchaga C., Goate A.M. (2014). Alzheimer’s disease genetics: From the bench to the clinic. Neuron.

[121] Fratiglioni L., Wang H.-X. (2000). Smoking and Parkinson’s and Alzheimer’s disease: Review of the epidemiological studies. Behav. Brain Res..

[122] Anstey K.J., von Sanden C., Salim A., O’Kearney R. (2007). Smoking as a Risk Factor for Dementia and Cognitive Decline: A Meta-Analysis of Prospective Studies. Am. J. Epidemiol..

[123] Cataldo J.K., Prochaska J.J., Glantz S.A. (2010). Cigarette Smoking is a Risk Factor for Alzheimer’s Disease: An Analysis Controlling for Tobacco Industry Affiliation. J. Alzheimers Dis..

[124] Rusanen M., Rovio S., Ngandu T., Nissinen A., Tuomilehto J., Soininen H., Kivipelto M. (2010). Midlife Smoking, Apolipoprotein E and Risk of Dementia and Alzheimer’s Disease: A Population-Based Cardiovascular Risk Factors, Aging and Dementia Study. Dement. Geriatr. Cogn. Disord..

[125] Merchant C., Tang M.-X., Albert S., Manly J., Stern Y., Mayeux R. (1999). The influence of smoking on the risk of Alzheimer’s disease. Neurology.

[126] Rusanen M., Kivipelto M., Quesenberry C.P., Zhou J., Whitmer R.A. (2011). Heavy Smoking in Midlife and Long-term Risk of Alzheimer Disease and Vascular Dementia. Arch. Intern. Med..

[127] Incalzi R.A., Gemma A., Marra C., Muzzolon R., Capparella O., Carbonin P. (1993). Chronic obstructive pulmonary disease: An original model of cognitive decline. Am. Rev. Respir. Dis..

[128] Dal Negro R.W., Bonadiman L., Bricolo F.P., Tognella S., Turco P. (2015). Cognitive dysfunction in severe chronic obstructive pulmonary disease (COPD) with or without Long-Term Oxygen Therapy (LTOT). Multidiscip. Respir. Med..

[129] Rusanen M., Ngandu T., Laatikainen T., Tuomilehto J., Soininen H., Kivipelto M. (2013). Chronic obstructive pulmonary disease and asthma and the risk of mild cognitive impairment and dementia: A population based CAIDE study. Curr. Alzheimer Res..

[130] Lutsey P.L., Chen N., Mirabelli M.C., Lakshminarayan K., Knopman D.S., Vossel K.A., Gottesman R.F., Mosley T.H., Alonso A. (2019). Impaired Lung Function, Lung Disease, and Risk of Incident Dementia. Am. J. Respir. Crit. Care Med..

[131] Akaike A., Takada-Takatori Y., Kume T., Izumi Y. (2010). Mechanisms of Neuroprotective Effects of Nicotine and Acetylcholinesterase Inhibitors: Role of α4 and α7 Receptors in Neuroprotection. J. Mol. Neurosci..

[132] Teaktong T., Graham A.J., Johnson M., Court J.A., Perry E.K. (2004). Selective changes in nicotinic acetylcholine receptor subtypes related to tobacco smoking: An immunohistochemical study. Neuropathol. Appl. Neurobiol..

[133] Egleton R.D., Abbruscato T. (2014). Drug Abuse and the Neurovascular Unit. Adv. Pharmacol..

[134] Abbruscato T.J., Lopez S.P., Mark K.S., Hawkins B.T., Davis T.P. (2002). Nicotine and Cotinine Modulate Cerebral Microvascular Permeability and Protein Expression of ZO-1 through Nicotinic Acetylcholine Receptors Expressed on Brain Endothelial Cells. J. Pharm. Sci..

[135] Moreno-Gonzalez I., Estrada L.D., Sanchez-Mejias E., Soto C. (2013). Smoking exacerbates amyloid pathology in a mouse model of Alzheimer’s disease. Nat. Commun..

[136] Wang X., Michaelis E.K. (2010). Selective neuronal vulnerability to oxidative stress in the brain. Front. Aging Neurosci..

[137] Durazzo T.C., Mattsson N., Weiner M.W. (2014). Alzheimer’s Disease Neuroimaging Initiative. Smoking and increased Alzheimer’s disease risk: A review of potential mechanisms. Alzheimers Dement..

[138] Falsetti L., Viticchi G., Zaccone V., Tarquinio N., Nobili L., Nitti C., Salvi A., Moroncini G., Silvestrini M. (2021). Chronic respiratory diseases and neurodegenerative disorders: A primer for the practicing clinician. Med. Princ. Pract..

[139] Zhang F., Niu L., Li S., Le W. (2019). Pathological impacts of chronic hypoxia on alzheimer’s disease. ACS Chem. Neurosci..

[140] Zhang X., Le W. (2010). Pathological role of hypoxia in Alzheimer’s disease. Exp. Neurol..

[141] Chow N., Bell R.D., Deane R., Streb J.W., Chen J., Brooks A., Van Nostrand W., Miano J.M., Zlokovic B.V. (2007). Serum response factor and myocardin mediate arterial hypercontractility and cerebral blood flow dysregulation in Alzheimer’s phenotype. Proc. Natl. Acad. Sci. USA.

[142] Viticchi G., Falsetti L., Vernieri F., Altamura C., Bartolini M., Luzzi S., Provinciali L., Silvestrini M. (2012). Vascular predictors of cognitive decline in patients with mild cognitive impairment. Neurobiol. Aging.

[143] Balucani C., Viticchi G., Falsetti L., Silvestrini M. (2012). Cerebral hemodynamics and cognitive performance in bilateral asymptomatic carotid stenosis. Neurology.

[144] Nelson A.R., Sweeney M.D., Sagare A.P., Zlokovic B.V. (2016). Neurovascular dysfunction and neurodegeneration in dementia and Alzheimer’s disease. Biochim. Biophys. Acta—Mol. Basis Dis..

[145] Halliday M.R., Pomara N., Sagare A.P., Mack W.J., Frangione B., Zlokovic B.V. (2013). Relationship Between Cyclophilin A Levels and Matrix Metalloproteinase 9 Activity in Cerebrospinal Fluid of Cognitively Normal Apolipoprotein E4 Carriers and Blood-Brain Barrier Breakdown. JAMA Neurol..

[146] Viticchi G., Falsetti L., Buratti L., Sajeva G., Luzzi S., Bartolini M., Provinciali L., Silvestrini M. (2017). Framingham Risk Score and the Risk of Progression from Mild Cognitive Impairment to Dementia. J Alzheimers Dis..

[147] Suri S., Mackay C.E., Kelly M.E., Germuska M., Tunbridge E.M., Frisoni G.B., Matthews P.M., Ebmeier K.P., Bulte D.P., Filippini N. (2015). Reduced cerebrovascular reactivity in young adults carrying the APOE ε4 allele. Alzheimers Dement..

[148] Buratti L., Balestrini S., Altamura C., Viticchi G., Falsetti L., Luzzi S., Provinciali L., Vernieri F., Silvestrini M. (2015). Markers for the risk of progression from mild cognitive impairment to Alzheimer’s disease. J. Alzheimers Dis..

[149] Viticchi G., Falsetti L., Burattini M., Zaccone V., Buratti L., Bartolini M., Moroncini G., Silvestrini M. (2020). Atrial Fibrillation on Patients with Vascular Dementia: A Fundamental Target for Correct Management. Brain Sci..

[150] Stanciu G.D., Ababei D.C., Bild V., Bild W., Paduraru L., Gutu M.M., Tamba B.-I. (2020). Renal Contributions in the Pathophysiology and Neuropathological Substrates Shared by Chronic Kidney Disease and Alzheimer’s Disease. Brain Sci..

[151] Ferrero J., Williams L., Stella H., Leitermann K., Mikulskis A., O’Gorman J., Sevigny J. (2016). First-in-human, double-blind, placebo-controlled, single-dose escalation study of aducanumab (BIIB037) in mild-to-moderate Alzheimer’s disease. Alzheimers Dement. Transl. Res. Clin. Interv..

[152] Tariq S., Barber P.A. (2018). Dementia risk and prevention by targeting modifiable vascular risk factors. J. Neurochem..

[153] Stephen R., Hongisto K., Solomon A., Lönnroos E. (2017). Physical Activity and Alzheimer’s Disease: A Systematic Review. J. Gerontol. Ser. A Biol. Sci. Med. Sci..

[154] Jeon S.Y., Byun M.S., Yi D., Lee J.-H., Ko K., Sohn B.K., Lee J.-Y., Ryu S.-H., Lee D.W., Shin S.A. (2020). Midlife Lifestyle Activities Moderate APOE ε4 Effect on in vivo Alzheimer’s Disease Pathologies. Front. Aging Neurosci..

[155] Nagai M., Hoshide S., Kario K. (2010). Hypertension and Dementia. Am. J. Hypertens..

[156] Alkasabera A., Onyali C.B., Anim-Koranteng C., Shah H.E., Ethirajulu A., Bhawnani N., Mostafa J.A. (2021). The Effect of Type-2 Diabetes on Cognitive Status and the Role of Anti-diabetes Medications. Cureus.

[157] Olmastroni E., Molari G., De Beni N., Colpani O., Galimberti F., Gazzotti M., Zambon A., Catapano A.L., Casula M. (2021). Statin use and risk of dementia or Alzheimer’s disease: A systematic review and meta-analysis of observational studies. Eur. J. Prev. Cardiol..

[158] Sheng M., Lu H., Liu P., Li Y., Ravi H., Peng S.-L., Diaz-Arrastia R., Devous M.D., Womack K.B. (2017). Sildenafil Improves Vascular and Metabolic Function in Patients with Alzheimer’s Disease. J. Alzheimers Dis..

[159] Sanders O. (2020). Sildenafil for the Treatment of Alzheimer’s Disease: A Systematic Review. J. Alzheimers Dis. Rep..

[160] Zuccarello E., Acquarone E., Calcagno E., Argyrousi E.K., Deng S.-X., Landry D.W., Arancio O., Fiorito J. (2020). Development of novel phosphodiesterase 5 inhibitors for the therapy of Alzheimer’s disease. Biochem. Pharmacol..

[161] Lattanzi S., Carbonari L., Pagliariccio G., Bartolini M., Cagnetti C., Viticchi G., Buratti L., Provinciali L., Silvestrini M. (2018). Neurocognitive functioning and cerebrovascular reactivity after carotid endarterectomy. Neurology.

[162] Buss S.S., Fried P.J., Pascual-Leone A. (2019). Therapeutic noninvasive brain stimulation in Alzheimer’s disease and related dementias. Curr. Opin. Neurol..

[163] Borgomaneri S., Battaglia S., Avenanti A., di Pellegrino G. (2021). Don’t Hurt Me No More: State-dependent Transcranial Magnetic Stimulation for the treatment of specific phobia. J. Affect. Disord..

[164] Borgomaneri S., Battaglia S., Sciamanna G., Tortora F., Laricchiuta D. (2021). Memories are not written in stone: Re-writing fear memories by means of non-invasive brain stimulation and optogenetic manipulations. Neurosci. Biobehav. Rev..

